# Right extrapleural completion pneumonectomy for fibrocavitary nontuberculous mycobacterial lung disease

**DOI:** 10.1002/ccr3.898

**Published:** 2018-01-11

**Authors:** Keiji Yamanashi, Norihito Okumura, Yohei Honda, Tomoaki Matsuoka

**Affiliations:** ^1^ Department of Thoracic Surgery Kurashiki Central Hospital Okayama Japan

**Keywords:** Bronchial stump fistula, extrapleural completion pneumonectomy, fibrocavitary nontuberculous mycobacterial lung disease

## Abstract

We report a case of a 76‐year‐old man with fibrocavitary nontuberculous mycobacterial (FC‐NTM) lung disease, who was successfully treated with right extrapleural completion pneumonectomy. Right extrapleural completion pneumonectomy with careful coverage of the bronchial stump might be effective in patients with FC‐NTM lung disease.

## Introduction

Nontuberculous mycobacterial (NTM) species are mycobacterial species other than those belonging to *Mycobacterium tuberculosis complex* and *M. leprae*. NTM species often cause chronic pulmonary infection refractory to antimicrobial therapy. The prevalence of NTM lung disease is increasing worldwide, and this disease can be either nodular/bronchiectatic or fibrocavitary (FC) radiographically 1. The prognosis of FC‐NTM lung disease is considerably poor because it commonly progresses quickly and can result in extensive cavitary lung destruction and respiratory failure 2. Surgical treatment for FC‐NTM lung disease is generally performed if other treatments are unsatisfactory.

Completion pneumonectomy is occasionally performed in FC‐NTM lung disease patients who have previously undergone pulmonary resection. Right completion pneumonectomy for FC‐NTM lung disease is considered a high‐risk operation 3, 4. Here, we report a case of FC‐NTM lung disease that was successfully treated with right extrapleural completion pneumonectomy.

## Case Presentation

**Figure 1:**
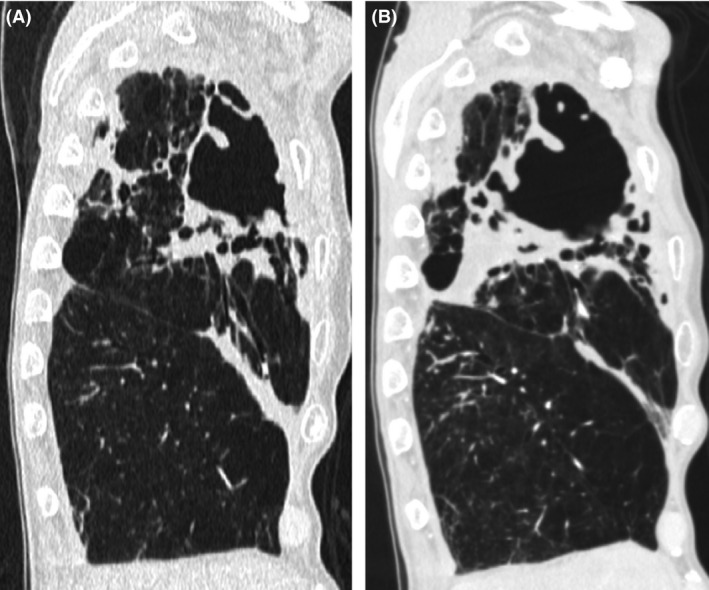
(A) Computed tomography image showing consolidation and large fibrocavitary changes in the right upper lobe. (B) Computed tomography image obtained four months later showing progression of consolidation and fibrocavitary changes.

**Figure 2:**
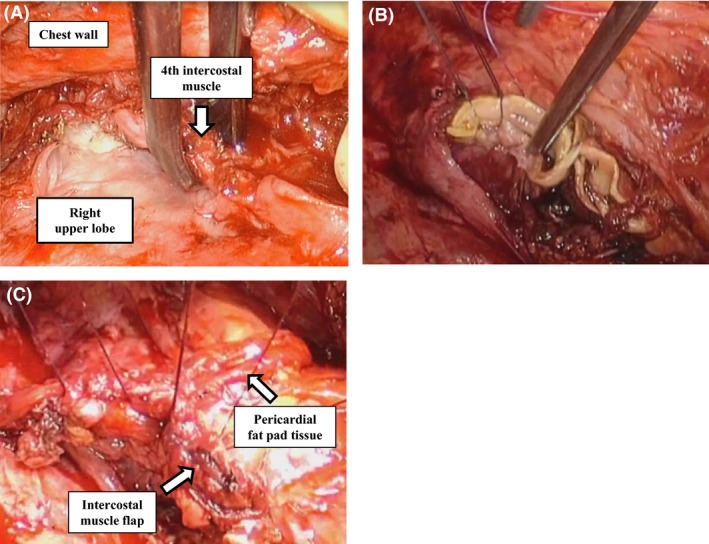
(A) Intraoperative image showing severe adhesions between the entire lung surface and the chest wall. (B, C) Image showing reinforcement of the bronchial stump with suturing (B), a pedicled intercostal muscle flap, and pericardial fat pad tissue (C).

A 76‐year‐old man with a history of right lower lobectomy with inferior mediastinal lymphadenectomy for squamous cell lung cancer (T1bN0M0) at 72 years of age was admitted to our hospital. He was receiving standard combination antibiotic treatment (clarithromycin, ethambutol, rifampin, and streptomycin) for FC‐NTM lung disease of the right upper and middle lobes involving *Mycobacterium intracellulare*. Although he had received combination antibiotic treatment for 4 months, he experienced persistent fever, weight loss, body mass index reduced to 16.1, malnutrition with an albumin level of 3.0 g/dL, and persistent positive sputum. A pulmonary function test showed percentage predicted vital capacity was 53.5%, forced expiratory volume in 1 sec (FEV_1_) 1.73 L, and FEV_1_/forced vital capacity ratio 91.1%. Maximum oxygen consumption was 12.2 mL/min/kg. Moreover, computed tomography showed progressing of consolidation and fibrocavitary changes (Fig. 1A and B). Therefore, right extrapleural completion pneumonectomy through right anteroaxillary thoracotomy and subsequent small lateral thoracotomy with preservation of the latissimus dorsi muscle were performed to remove the infection focus. A pedicled latissimus dorsi muscle flap could be used as a buttress for the bronchial stump on occurrence of a bronchial stump fistula. Severe adhesions were observed over the entire surface of the lung and chest wall owing to previous surgery and his FC‐NTM lung disease (Fig. 2A), and we dissected almost all adhesions extrapleurally with resection of parietal pleura and mediastinal pleura. Extrapericardial dissection was performed, and the right superior pulmonary vein was isolated. The right main bronchus was closed with a TLH‐30 stapler (Ethicon, Inc., Somerville, NJ), and the bronchial stump was sutured with 3‐0 Vicryl (Ethicon, Inc.) and reinforced with a pedicled intercostal muscle flap and pericardial fat pad tissue (Fig. 2B and C). Postoperatively, aggressive nutritional support was continued with oral supplements and the same combination antibiotic treatment was maintained. The postoperative course was uneventful, and he was discharged from the hospital 1 month after surgery. Chest radiography performed 4 months after discharge showed a slight decrease in the niveau and pleural effusion, although he had no symptoms and no inflammatory response on hematological assessment. A pedicled latissimus dorsi muscle flap was used as a buttress for the bronchial stump because late bronchial stump fistula was suspected.

## Outcome and Follow‐up

He is alive 12 months after the procedure, with no sign of recurrence of FC‐NTM lung disease or a bronchial stump fistula.

## Discussion

There are no published patient selection criteria of surgical treatment for NTM lung disease 1. Van Ingen et al. reported timing of surgical treatment for NTM lung disease is essential to prevent disease progression to a stage where safe and effective surgery is no longer possible, because development of cavitary lesions in the contralateral lung, deterioration of patient condition, and acquisition of further drug resistance may render adjunctive surgery and postoperative chemotherapy ineffective 5. Moreover, Sherwood et al. reported the indications for surgical treatment of FC‐NTM lung disease include failure to thrive, persistent localized cavitary disease or destroyed lung, persistent sputum positivity, hemoptysis, bronchial stump fistula, and bronchial stenosis 4. In the present case, although the physical condition of the patient was eligible for surgery, we hesitated to perform completion pneumonectomy because this procedure has been reported to have high mortality and morbidity rates 4 and a high rate of bronchial stump fistula development 3, 4. However, our patient had constitutional symptoms and there was a risk of contamination of healthy contralateral lung parenchyma. Therefore, we decided to perform right extrapleural completion pneumonectomy before further respiratory deterioration.

A previous report recommended tailored suture closure of the bronchial stump and use of the omentum for coverage in right completion pneumonectomy for NTM lung disease 4. In the present case, the use of the omentum was avoided because of the patient's history of abdominal surgery. The bronchial stump was sutured after stapling and was reinforced with a pedicled intercostal muscle flap and pericardial fat pad tissue. Moreover, a pedicled latissimus dorsi muscle flap was used because a late bronchial stump fistula was suspected. It is possible that these careful procedures led to the satisfactory outcome in the present case. Consequently, he was discharged home independently and is alive 12 months after the procedure, with no sign of recurrence of FC‐NTM lung disease, a bronchial stump fistula, or the other complications, although the prognosis of FC‐NTM lung disease is considerably poor 2. Therefore, surgical treatment for NTM lung disease was useful in terms of overall survival and quality of life in the present case.

Antibiotic treatment failure is considered if there has been no response after 6 months of appropriate therapy, and patients commonly receive preoperative antibiotic treatment over 6 months 1, 6. However, long duration of antibiotic treatment might worsen the patient's condition if NTM lung disease progresses. In the present case, NTM lung disease progressed rapidly, constitutional symptoms were noted, and there was a risk of contamination of the healthy contralateral lung; therefore, he received antibiotic treatment for only 4 months and urgently underwent surgical treatment, which might have led to his satisfactory outcome. In conclusion, we reported a case of FC‐NTM lung disease that was successfully treated with right extrapleural completion pneumonectomy. Right extrapleural completion pneumonectomy with careful coverage of the bronchial stump might be effective in patients with FC‐NTM lung disease.

## Authorship

KY: drafted the manuscript. NO: was a major contributor in writing the manuscript. YH and TM: participated in data collection.

## Conflict of Interest

None declared.
